# Phylogenic analysis and forensic genetic characterization of Chinese Uyghur group via autosomal multi STR markers

**DOI:** 10.18632/oncotarget.17992

**Published:** 2017-05-18

**Authors:** Xiaoye Jin, Yuanyuan Wei, Jiangang Chen, Tingting Kong, Yuling Mu, Yuxin Guo, Qian Dong, Tong Xie, Haotian Meng, Meng Zhang, Jianfei Li, Xiaopeng Li, Bofeng Zhu

**Affiliations:** ^1^ Key Laboratory of Shaanxi Province for Craniofacial Precision Medicine Research, College of Stomatology, Xi'an Jiaotong University, Xi'an, 710004, Shaanxi, PR China; ^2^ Clinical Research Center of Shaanxi Province for Dental and Maxillofacial Diseases, College of Stomatology, Xi'an Jiaotong University, Xi'an, 710004, Shaanxi, PR China; ^3^ College of Medicine and Forensics, Xi'an Jiaotong University Health Science Center, Xi'an, 710061, Shaanxi, PR China; ^4^ Department of Biochemistry, Preclinical Medicine College, Xinjiang Medical University, Urumqi, 830011, Xinjiang, PR China; ^5^ Department of Forensic Genetics, School of Forensic Medicine, Southern Medical University, Guangzhou, 510515, Guangdong, PR China; ^6^ Department of Orthopaedics, Ankang City Central Hospital, Ankang, 725000, Shaanxi, PR China; ^7^ Xi'an Jiaotong University, Xi'an, 710049, Shaanxi, PR China; ^8^ Department of Ultrasound, The Second Affiliated Hospital of Xi'an Jiaotong University, Xi'an, 710004, Shaanxi, PR China

**Keywords:** short tandem repeats (STRs), inter-population differentiation, Uyghur ethnic group, phylogenic analysis, PCA

## Abstract

We investigated the allelic frequencies and forensic descriptive parameters of 23 autosomal short tandem repeat loci in a randomly selected sample of 1218 unrelated healthy Uyghur individuals residing in the Xinjiang Uyghur Autonomous Region, northwest China. A total of 281 alleles at these loci were identified and their corresponding allelic frequencies ranged from 0.0004 to 0.5390. The combined match probability and combined probability of exclusion of all loci were 5.192 × 10^−29^ and 0.9999999996594, respectively. The results of population genetic study manifested that Uyghur had close relationships with those contiguous populations, such as Xibe and Hui groups. In a word, these autosomal short tandem repeat loci were highly informative in Uyghur group and the multiplex PCR system could be used as a valuable tool for forensic caseworks and population genetic analysis.

## INTRODUCTION

The Uyghur ethnic group, the fourth largest ethnic minority of China, primarily lives in northwest China’s Xinjiang Uyghur Autonomous Region. Previous population genetic studies [1–3] indicated that Uyghurs possess mixed anthropometric and genetic traits of both Europeans and Central Asians. Therefore, the genetic data analysis of more markers in Uyghur group will be of benefit to shed light on its genetic relationships with other populations.

Short tandem repeats (STRs) have been publicly recognized as vital genetic markers in forensic sciences. In order to achieve better performances in forensic applications, especially in some mutation events of parentage testing, we need more genetic markers with high polymorphisms. Meantime, we should obtain population genetic data of these markers as much as possible before putting them into actual forensic cases. In this study, we investigated the allelic frequencies and forensic descriptive parameters of autosomal multi STR loci in Uyghur group using HuaXia Platinum PCR Amplification system, which included 23 autosomal STR loci and two sex associated markers (Y-chromosome insertion and deletion and Amelogenin). Furthermore, we evaluated genetic relationships between studied Uyghur group and other reference populations [4–17] from the same or different regions based on 14 shared STR loci including D8S1179, D21S11, D7S820, CSF1PO, TH01, D13S317, D16S539, D2S1338, D19S433, vWA, TPOX, D18S51, D5S818 and FGA loci.

## RESULTS

### Allelic frequencies and forensic descriptive parameters

The results of allelic frequencies and forensic descriptive parameters for 23 STR loci were presented in Table 1 and Table 2, respectively. A total of 281 alleles were observed in Uyghur group with their corresponding frequencies ranging from 0.0004 to 0.5390. For Hardy-Weinberg equilibrium (HWE) test, twenty-two of 23 STR loci were observed to exhibit HWE after Bonferroni correction (*p* = 0.05/23≈0.0022); only D3S1358 violated Hardy-Weinberg equilibrium. The match of probability (MP) ranged from 0.0123 at Penta E locus to 0.2003 at TPOX locus with the average of 0.0692. The probability of exclusion (PE) ranged from 0.2862 at TPOX locus to 0.8102 at Penta E locus with the average of 0.5969. The mean values of discrimination power (DP), polymorphic information content (PIC), observed heterozygosity (Ho) and expected heterozygosity (He) were 0.9380, 0.7776, 0.7957 and 0.8048, respectively; and the highest values of DP, PIC, Ho and He were also found at Penta E locus, the least observed at TPOX locus. The combined match probability (CMP) and combined probability of exclusion (CPE) of 23 STR loci were 5.192×10^-29^ and 0.9999999996594, respectively.

**Table 1:** Allelic frequencies for 23 autosomal STR loci of Uyghur group in the Xinjiang Uyghur Autonomous Region, northwest China

Allele	D19S433	Allele	Penta E	Allele	CSF1PO	Allele	D2S441	Allele	D12S391	Allele	D18S51	Allele	D21S11	Allele	D10S1248
10	0.0004	5	0.0283	8	0.0008	9	0.0004	15	0.0111	10	0.0041	26	0.0012	11	0.0177
11	0.0021	6	0.0008	9	0.0287	9.1	0.0049	16	0.0066	11	0.0131	27	0.0082	12	0.0480
12	0.0534	7	0.0661	10	0.3025	10	0.2438	17	0.1145	12	0.0538	28	0.0866	13	0.2779
12.2	0.0062	8	0.0086	11	0.2553	10.1	0.0029	17.3	0.0053	13	0.1704	28.2	0.0012	14	0.2574
13	0.2360	9	0.0033	12	0.3264	11	0.3970	18	0.2184	14	0.2147	29	0.2274	15	0.2393
13.2	0.0394	10	0.0472	13	0.0657	11.3	0.0259	18.2	0.0012	15	0.1576	29.2	0.0066	16	0.1252
14	0.2828	11	0.1186	14	0.0177	12	0.1117	18.3	0.0140	16	0.1314	30	0.2529	17	0.0259
14.2	0.0681	12	0.1215	15	0.0029	12.3	0.0004	19	0.1995	17	0.0948	30.2	0.0222	18	0.0082
15	0.1096	13	0.0739	**Allele**	**D3S1358**	13	0.0197	19.3	0.0021	18	0.0546	30.3	0.0029	19	0.0004
15.2	0.1355	14	0.0759	12	0.0004	14	0.1819	20	0.1342	19	0.0472	31	0.0710	**Allele**	**D5S818**
16	0.0361	15	0.0899	13	0.0004	15	0.0111	21	0.1305	20	0.0218	31.2	0.1088	7	0.0086
16.2	0.0213	15.3	0.0008	14	0.0583	16	0.0004	22	0.0837	20.1	0.0004	32	0.0209	8	0.0033
17	0.0041	16	0.0833	15	0.3034	**Allele**	**D6S1043**	23	0.0386	21	0.0156	32.2	0.1322	9	0.0669
17.2	0.0049	16.4	0.0012	16	0.3112	7	0.0008	24	0.0279	22	0.0082	33	0.0008	10	0.1199
**Allele**	**FGA**	17	0.0928	17	0.2213	8	0.0021	25	0.0115	23	0.0041	33.2	0.0517	11	0.3555
17	0.0053	18	0.0702	18	0.0944	9	0.0074	26	0.0008	24	0.0070	34.2	0.0053	12	0.2956
18	0.0074	19	0.0353	19	0.0090	10	0.0123	**Allele**	**D16S539**	25	0.0004	**Allele**	**vWA**	13	0.1416
19	0.0435	20	0.0378	20	0.0016	11	0.2410	8	0.0205	26	0.0008	13	0.0045	14	0.0082
20	0.0718	21	0.0094	**Allele**	**D7S820**	12	0.1605	9	0.2007	**Allele**	**D1S1656**	14	0.1416	15	0.0004
21	0.1470	22	0.0177	7	0.0070	13	0.1141	10	0.1092	8	0.0037	15	0.0636	**Allele**	**D8S1179**
21.2	0.0008	23	0.0057	8	0.2077	14	0.1170	11	0.2623	9	0.0004	16	0.2504	7	0.0004
22	0.1519	24	0.0057	9	0.0825	15	0.0156	12	0.2549	10	0.0070	17	0.2607	8	0.0049
22.2	0.0123	25	0.0045	9.1	0.0008	16	0.0021	13	0.1326	11	0.0989	18	0.1868	9	0.0078
23	0.1704	26	0.0012	10	0.2102	17	0.0328	14	0.0189	12	0.0784	19	0.0747	10	0.0920
23.2	0.0041	**Allele**	**D2S1338**	11	0.2833	18	0.1158	15	0.0008	13	0.0628	20	0.0168	11	0.0850
24	0.2065	13	0.0021	11.1	0.0008	19	0.1252	**Allele**	**Penta D**	14	0.1010	21	0.0008	12	0.1051
24.2	0.0008	15	0.0004	12	0.1769	20	0.0464	6	0.0115	15	0.2455	**Allele**	**D22S1045**	13	0.2759
25	0.1260	16	0.0103	13	0.0283	21	0.0053	7	0.0148	15.3	0.0197	11	0.2533	14	0.1970
25.2	0.0012	17	0.0977	14	0.0025	21.3	0.0004	8	0.0250	16	0.1880	12	0.0029	15	0.1527
26	0.0419	18	0.1076	**Allele**	**D13S317**	22	0.0012	9	0.2853	16.3	0.0287	13	0.0049	16	0.0718
27	0.0062	19	0.1917	8	0.1486	**Allele**	**TPOX**	10	0.1375	17	0.0628	14	0.0489	17	0.0062
28	0.0029	20	0.1400	9	0.1047	7	0.0004	11	0.1979	17.3	0.0447	15	0.3231	18	0.0012
**Allele**	**TH01**	21	0.0259	10	0.1219	8	0.5390	12	0.1572	18	0.0111	16	0.2172		
6	0.1851	22	0.0476	11	0.2956	9	0.0874	13	0.1137	18.3	0.0328	17	0.1273		
7	0.2804	23	0.1868	12	0.2114	10	0.0472	14	0.0411	19	0.0008	18	0.0209		
8	0.1030	24	0.1002	13	0.0862	11	0.2759	15	0.0135	19.3	0.0111	19	0.0016		
9	0.2989	25	0.0673	14	0.0300	12	0.0493	16	0.0025	20.3	0.0025				
9.3	0.1256	26	0.0205	15	0.0016	13	0.0008								
10	0.0070	27	0.0021												

**Table 2:** Forensic descriptive parameters for 23 autosomal STR loci of Uyghur group in the Xinjiang Uyghur Autonomous Region, northwest China

Loci	MP	DP	PIC	PE	Ho	He	*p*
CSF1PO	0.1207	0.8793	0.6831	0.4814	0.7332	0.7313	0.3023
D10S1248	0.0801	0.9199	0.7458	0.5161	0.7537	0.7802	0.1110
D12S391	0.0384	0.9616	0.8380	0.7155	0.8604	0.8545	0.3008
D13S317	0.0605	0.9395	0.7868	0.6386	0.8210	0.8117	0.8202
D16S539	0.0710	0.9290	0.7649	0.5668	0.7824	0.7956	0.7072
D18S51	0.0319	0.9681	0.8503	0.6671	0.8358	0.8646	0.0047
D19S433	0.0509	0.9491	0.8017	0.6153	0.8087	0.8230	0.3590
D1S1656	0.0309	0.9691	0.8529	0.7057	0.8555	0.8658	0.1503
D21S11	0.0442	0.9558	0.8200	0.6480	0.8259	0.8387	0.8892
D22S1045	0.0917	0.9083	0.7277	0.5133	0.7521	0.7653	0.0477
D2S1338	0.0318	0.9682	0.8559	0.7204	0.8629	0.8696	0.0042
D2S441	0.1082	0.8918	0.6962	0.4841	0.7348	0.7362	0.5120
D3S1358	0.1035	0.8965	0.7075	0.4679	0.7250	0.7498	0.0013
D5S818	0.1048	0.8952	0.7075	0.4882	0.7373	0.7472	0.2904
D6S1043	0.0374	0.9626	0.8409	0.6928	0.8489	0.8567	0.2279
D7S820	0.0741	0.9259	0.7621	0.5924	0.7964	0.7935	0.7715
D8S1179	0.0519	0.9481	0.8089	0.6544	0.8292	0.8297	0.0235
FGA	0.0371	0.9629	0.8427	0.7074	0.8563	0.8587	0.3969
Penta D	0.0564	0.9436	0.7969	0.6292	0.8161	0.8200	0.3302
Penta E	0.0123	0.9877	0.9152	0.8102	0.9072	0.9207	0.2037
TH01	0.0870	0.9130	0.7348	0.5332	0.7635	0.7714	0.2223
TPOX	0.2003	0.7997	0.5678	0.2862	0.5961	0.6211	0.0040
vWA	0.0665	0.9335	0.7764	0.5954	0.7980	0.8045	0.9422

MP: match of probability; DP: discrimination power; PIC: polymorphic information content; PE: probability of exclusion; Ho: observed heterozygosity; He: expected heterozygosity; *p*: probability values for Hardy-Weinberg equilibrium tests.

### Comparisons of inter-population genetic differentiations and genetic distances

As shown in Table 3, the results of population differentiation comparisons (*p*-value) were obtained with the method of analysis of molecular variance (AMOVA). After applying Bonferroni correction to multiple tests (*p =* 0.05/238≈0.00021), the least differentiations were found between Uyghur group and Uyghur1, Hui and Xibe groups at the same 14 loci, with significant differences at 0, 1 and 3 loci, respectively. By contrast, more significant differences were observed between Uyghur group and African American and Caucasian populations, with significant differences at 13 loci. Among all the compared loci, the most population diversity locus was found at TH01 with significant difference between Uyghur and 16 reference populations, the least found at TPOX with only 2 reference populations.

**Table 3:** The results of inter-population differentiations (p-value) between Uyghur group and other previously published populations using the AMOVA method

Populations	D8S1179	D21S11	D7S820	CSF1PO	TH01	D13S317	D16S539	D2S1338	D19S433	vWA	TPOX	D18S51	D5S818	FGA
Spanish	0.8065	0.0039	0.0049	0.5347	**0.0000**	0.2600	0.0029	**0.0000**	**0.0000**	0.5855	0.1408	**0.0000**	0.0088	**0.0000**
Mexican	**0.0000**	0.0068	**0.0000**	0.0068	**0.0000**	**0.0000**	**0.0000**	**0.0000**	**0.0000**	0.0010	0.0068	**0.0000**	0.0068	**0.0000**
Portuguese	**0.0000**	**0.0000**	**0.0000**	0.1398	**0.0000**	**0.0000**	**0.0000**	**0.0000**	**0.0000**	0.0020	0.1290	**0.0000**	**0.0000**	**0.0000**
African American	**0.0000**	**0.0000**	**0.0000**	**0.0000**	**0.0000**	**0.0000**	0.0059	**0.0000**	**0.0000**	**0.0000**	**0.0000**	**0.0000**	**0.0000**	**0.0000**
Caucasian	**0.0000**	**0.0000**	**0.0000**	**0.0000**	**0.0000**	**0.0000**	**0.0000**	**0.0000**	**0.0000**	**0.0000**	0.1545	**0.0000**	**0.0000**	**0.0000**
Hispanics	0.0108	0.8954	**0.0000**	0.0108	**0.0000**	**0.0000**	0.0440	0.0010	**0.0000**	**0.0000**	0.0235	**0.0000**	0.0039	**0.0000**
Asian	0.1848	0.0059	0.0186	0.0078	**0.0000**	0.1144	**0.0000**	0.7801	0.3275	**0.0000**	1.0000	0.7771	**0.0000**	0.0020
Uyghur1	0.0205	0.7967	0.8084	0.0244	0.9208	0.1105	0.6725	0.1154	0.9296	0.5582	0.3226	0.1075	0.0420	0.8798
Xibe	0.9629	0.0059	0.0108	**0.0000**	**0.0000**	**0.0000**	0.0049	0.0098	0.1105	0.0020	0.4106	0.0557	0.1163	0.0010
Tibetan	**0.0000**	**0.0000**	0.0020	**0.0000**	**0.0000**	**0.0000**	**0.0000**	**0.0000**	**0.0000**	0.0010	**0.0000**	**0.0000**	**0.0000**	**0.0000**
She	**0.0000**	**0.0000**	**0.0000**	**0.0000**	**0.0000**	**0.0000**	0.6637	**0.0000**	**0.0000**	**0.0000**	0.1633	0.2239	0.0186	**0.0000**
Yi	**0.0000**	**0.0000**	**0.0000**	0.0029	**0.0000**	**0.0000**	0.0733	0.0029	0.0352	**0.0000**	0.8553	0.0020	0.0049	0.0020
Hui	0.0196	0.1193	0.2199	0.5288	**0.0000**	0.0381	0.0821	0.0176	0.3949	0.1818	0.0303	0.1672	0.8182	0.2356
Beijing Han	0.3695	**0.0000**	**0.0000**	0.0020	**0.0000**	**0.0000**	0.0010	**0.0000**	0.0225	**0.0000**	0.3001	0.0059	0.0068	0.0029
Guangdong Han	0.0010	0.2014	**0.0000**	0.1310	**0.0000**	**0.0000**	0.2874	0.9697	**0.0000**	**0.0000**	0.3089	0.2024	0.0117	**0.0000**
Shaanxi Han	0.0352	0.0059	**0.0000**	0.0088	**0.0000**	**0.0000**	0.0450	**0.0000**	**0.0000**	**0.0000**	0.0039	0.3451	0.0039	**0.0000**
Henan Han	0.0049	**0.0000**	**0.0000**	**0.0000**	**0.0000**	**0.0000**	**0.0000**	**0.0000**	**0.0000**	**0.0000**	0.0020	0.0020	**0.0000**	**0.0000**

Notes: The numbers in bold mean statistically significant after Bonferroni correction (*p* = 0.05/238≈0.00021).

The pairwise fixation index (*Fst*) genetic distances between Uyghur group and other 17 reference populations were generated by the Genepop v4.0.10 [18] based on 14 overlapping STR loci which were shown in Supplementary Table 1. The close genetic distances were found between two Uyghur groups (0.0009), followed by Hui (0.0038) and Xibe (0.0043) groups; and the longest distance was observed between Uyghur and Mexican (0.0225) group.

### Multidimensional scaling and principal component analysis

**Figure 1:**
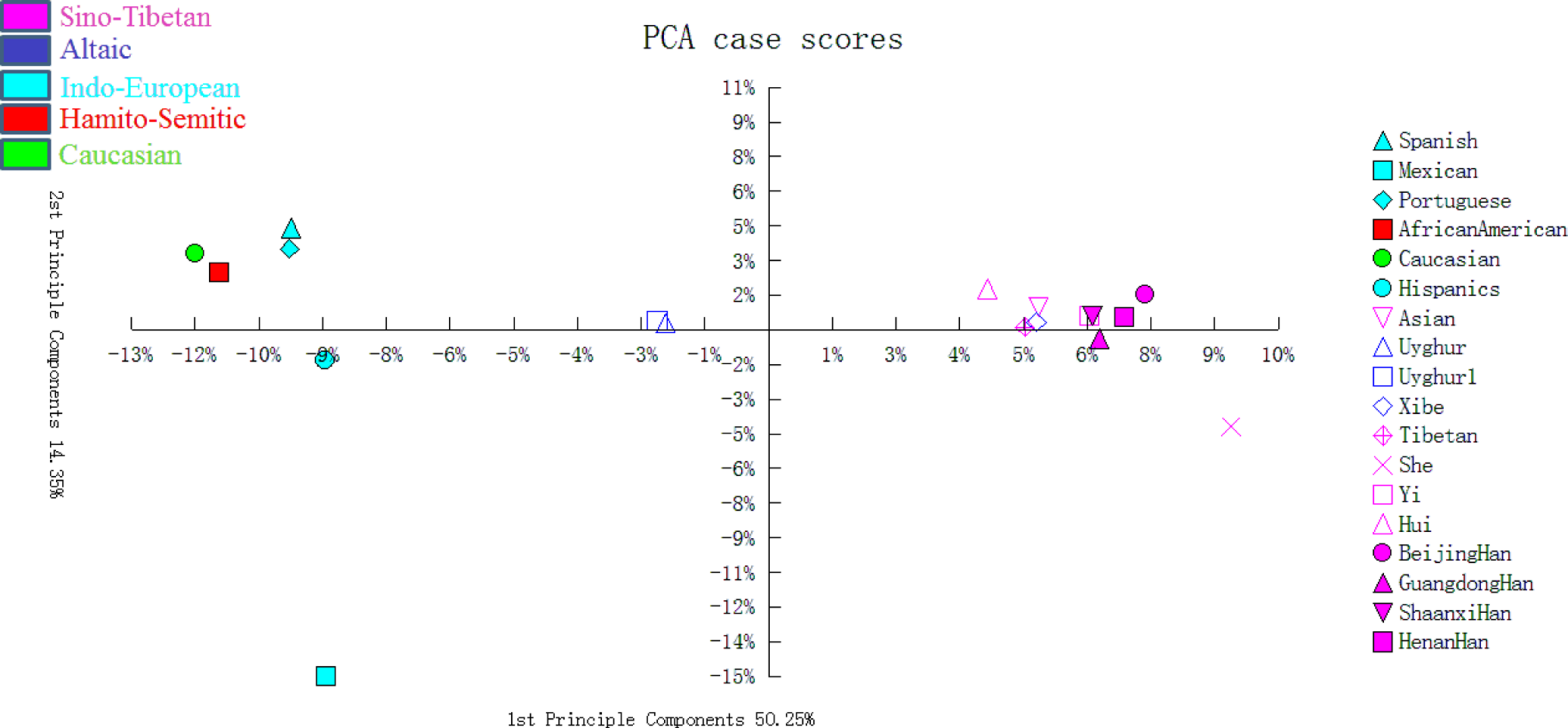
The PCA of Uyghur and other 17 reference populations based on the 14 STRs

**Figure 2:**
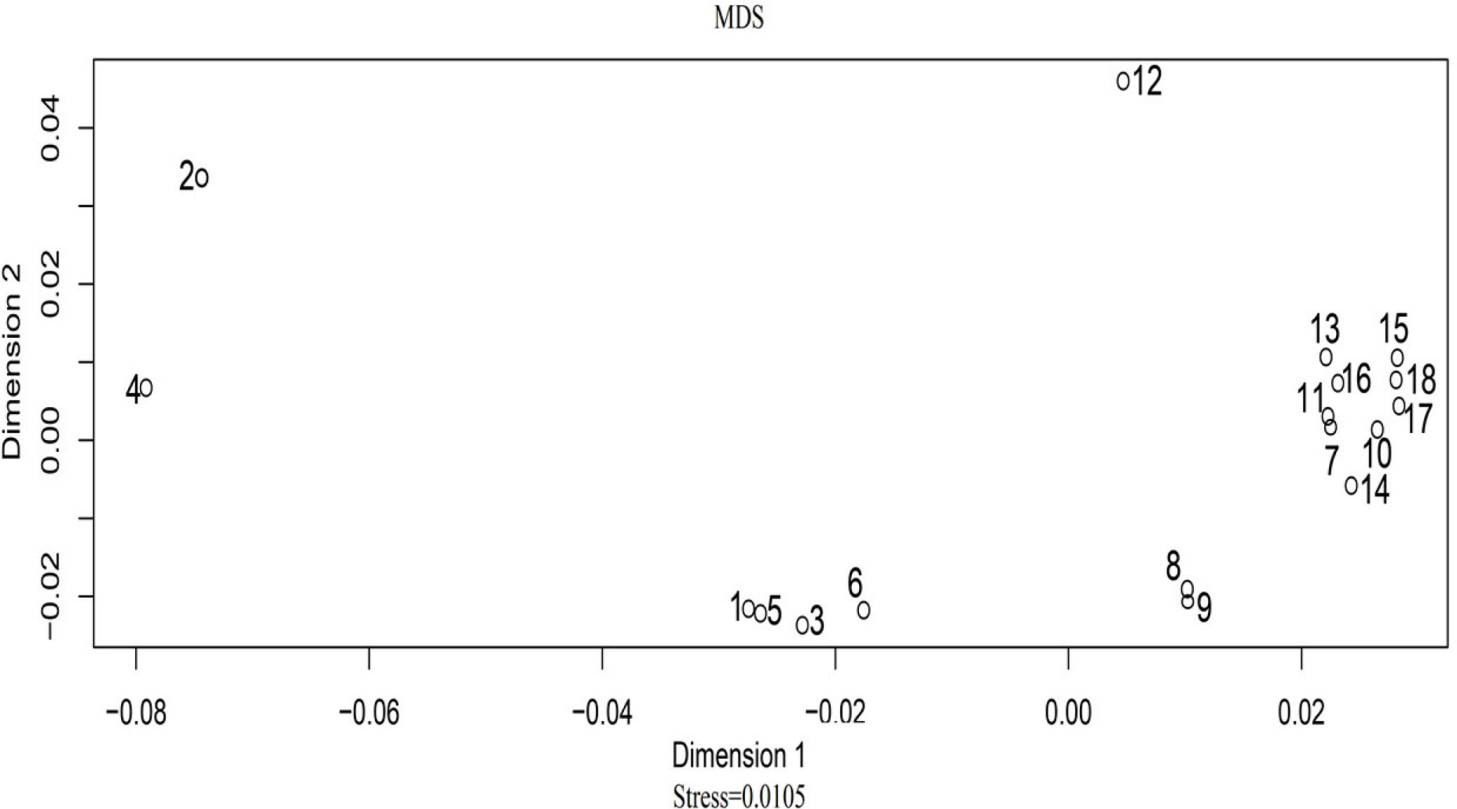
The MDS plot based on *Fst* distances across 18 populations The annotations of the above-mentioned number are listed as follows: 1, Spanish; 2, Mexican; 3, Portuguese; 4, African American; 5, Caucasian; 6, Hispanics; 7, Asian; 8, Uyghur; 9, Uyghur1; 10, Xibe; 11, Tibetan; 12, She; 13,Yi; 14, Hui; 15, Beijing Han; 16, Guangdong Han; 17, Shaanxi Han; 18, Henan Han.

A principal component analysis (PCA) plot was drawn using allelic frequencies of 14 shared loci of Uyghur group and 17 previously published populations by MVSP v3.1 software [19]. As demonstrated in Figure 1, four conglomerate groups were obviously observed: the first group consisting of Asian and nine Chinese populations including Beijing Han, Henan Han, Shaanxi Han, Guangdong Han, Hui, Tibetan, She, Yi and Xibe populations located in the right part of the graph; the second group including Spanish, Portuguese, Caucasian, and African American populations located in the top left corner; the third one, Hispanics and Mexican group, found to be positioned in the lower left part; the last group of two Uyghur groups clustering together located in the middle part. As shown in Figure 2, a multidimensional scaling (MDS) plot of eighteen populations was illustrated based on genetic distances (*Fst* values) using the ‘R’ package (http://www.r-tutor.com/category/r-packages). The similar population distributions were observed in the MDS plot: Chinese populations and Asian population were located in the right part; Mexican and African American were located in the left part; Spanish, Caucasian, Portuguese and Hispanics were located in the middle of the plot.

### Phylogenic reconstructions

**Figure 3:**
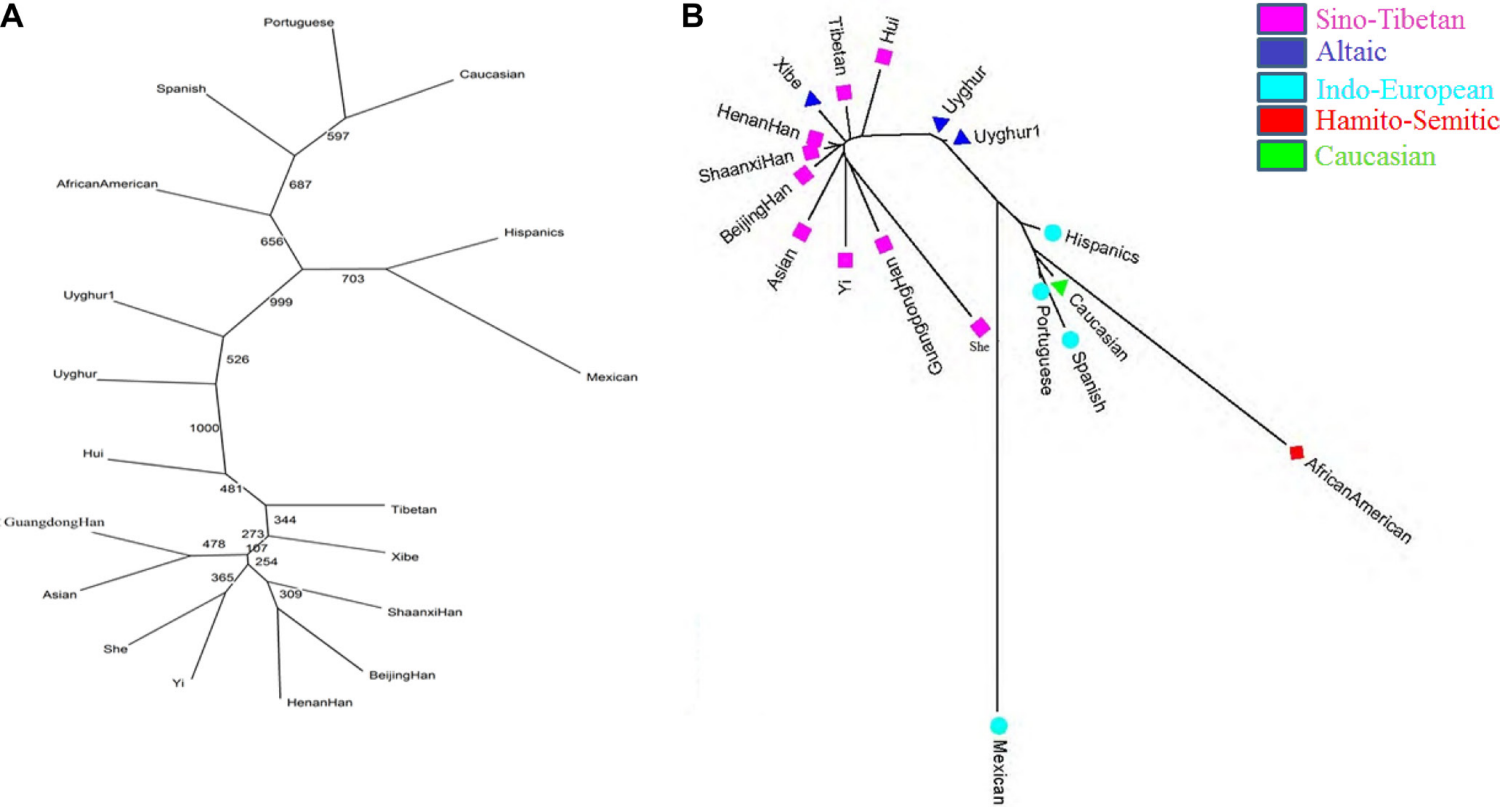
Phylogenic trees among 18 populations based on allelic frequencies by PHYLIP v3.6 software and *D*_*A*_ distances by MEGA v5 software, respectively

By using neighbor-joining method, two phylogenic trees were constructed by the software PHYLIP v3.6 (Figure 3A) and MEGA v5 (Figure 3B), respectively. The similar results which were obtained from two phylogenic trees depicted three clusters. And the first cluster consisted of Hui, Tibetan, Xibe, Shaanxi Han, Beijing Han, Henan Han, Yi, She, Asian and Guangdong Han; the second clade was shared by two Chinese Uyghur groups; the last comprised Mexican, Hispanics, African American, Spanish, Portuguese and Caucasian populations.

## DISCUSSION

### Forensic parameter analysis

HWE announces that a large random-mating population without selecting, mutating or migrating is thought to be in HWE. For populations in HWE, allelic frequencies and genotype frequencies usually remain constant from generation to generation [20–21]. Since HWE was influenced by multiple factors such as genetic drift, gene flowing and so on, it states an ideal condition with respect to the studied population. In the present study, only D3S1358 locus was observed to deviate from the HWE. A possible explanation for the deviation might result from population substructure. Since the blood samples we collected were from the whole territory of Xinjiang which comprises multiple ethnic minorities, individuals from different regions may form population substructures because of their geographical isolations. At 23 STR loci, the Penta E locus possessed the greatest values of PD and PE, whereas the TPOX locus owned the lowest. Previous studies [22–23] also found the similar results, which indicated Penta E was the most valuable locus and TPOX was a lower polymorphic locus than other STR loci in forensic cases. Therefore, we should screen more highly polymorphic loci so as to obtain better system effectiveness in subsequent forensic DNA and population genetic studies. The CMP and CPE could be regarded as indicators to evaluate efficiency of genetic markers in forensic application. In this study, the CMP and CPE obtained from the studied Uyghur group were 5.192 × 10^–29^ and 0.9999999996594, respectively, which indicated the panel could be a robust and valid tool for individual identification and parentage testing in forensic caseworks.

### Analysis of inter-population differentiations and genetic distances

If the loci with significant differences in allelic frequencies between pairwise populations are too many, it means that their genetic relationships are long distance and vice versa. Comparisons of genetic differentiations between Uyghur group and other reference populations based on 14 overlapping STR loci indicated the Uyghur had closer genetic relationships with some Chinese populations than those from different continents. By investigating inter-population differentiations based on allelic frequencies using AMOVA method, the two Uyghur groups showed no significant differences at these loci, and the significant differences were found between the studied Uyghur and Hui group at only TH01 locus; Xibe group at CSF1PO, TH01, D13S317 loci, revealing the short distances between Uyghur and Hui, Xibe groups. Deng [24] et al. reported the similar result that significant difference between Hui and Uyghur group was only observed at TH01 locus at the same 14 STR loci. Furthermore, the TH01 locus showed the most population diversities in the current study, which was also reported by Meng et al [9]; and the locus would have contributed to study the genetic differentiations among different populations.

*Fst* is a measure of genetic differentiation between compared populations which is used frequently in population genetics. The pairwise populations whose *Fst* is small usually show similar allelic frequency distributions; on the contrary, they show greatly different. In this study, the relative small *Fst* values were observed between Uyghur group with its adjacent populations such as Hui and Xibe groups, which indicated they had tight genetic relationships.

### Phylogenic analysis

The genetic relationships between Uyghur group and 17 reference populations were revealed by the PCA and MDS figures based on allelic frequencies of the same 14 STR and population pairwise *Fst* values, respectively. The results of PCA and MDS analysis indicated that the populations with closer geographical distances had more intimate relationships. Besides, results obtained once again showed that Uyghur is a Eurasian population. In order to further evaluate genetic relationships among these populations, we constructed two phylogenic trees. The results obtained from phylogenic trees were basically in agreement with above genetic relationship analysis. In addition, previous studies also reported the similar results as following. Shen et al. constructed a phylogenic tree between Uyghur group and other reported populations based on allelic frequencies of HLA-B locus, revealing that Uyghur group had close genetic relationships with the Xibe and Hui groups [25]. A dendrogram which was conducted using allelic frequencies of four VNTRs and a STR locus demonstrated that Uyghur, Hui and other three Asian groups shared the same branch [26]. Bian et al. reported that Hui was most closely related to Uyghur with the method of pairwise *Rst* at 17 Y-STR loci [27]. The close relationship between Uyghur group and Hui group may be attributed to the analogous historical backdrops. On the one hand, both the ethnic minorities mainly located in northwestern China, an important zone crossed by the famous Silk Road. Since the convenient advantages of their geographical locations, both may have undergone extensive interactions which led to their similar genetic pattern. On the other hand, Uyghur and Hui group both have diverse origins, especially Hui ethnic group. Previous study [28] indicated that Huis in northwest China were derived from the convergence of Mongol, Turkic, Iranian and other Central Asia settlers including Han people and Uighurs. To sum up, both nationalities may have intimate blood relationships.

## MATERIALS AND METHODS

### Population samples and DNA extraction

We gathered blood samples from 1218 unrelated healthy individuals of Uyghur ethnic minority in northwest China’s Xinjiang Uyghur Autonomous Region after receiving their written informed consents. Each individual whose family has been living in the region for at least three generations didn’t intermarriage with other ethnic groups. All experiment procedures were in agreement with the ethical committee of Xi'an Jiaotong University Health Science Center, China. Under the instructions of Walsh et al, we obtained genomic DNA of each sample with the Chelex-100 method [29].

### PCR and DNA typing

All loci were co-amplified using HuaXia Platinum PCR Amplification kit in GeneAmp 9700 PCR system (Applied Biosystems, Foster City, USA) under the producer’s specification. Thermal cycler conditions were as described below: pre-denaturation at 95°C for 1 min, followed by 27 cycles of 94°C for 3 s, 59°C for 16 s, 65°C for 29 s, and a final extension for 5 min at 60°C. The whole PCR reaction could be finished in 1 hour. Separation of amplified products was performed by capillary electrophoresis on the ABI 3130xl Genetic Analyzer (Applied Biosystems, Foster City, CA, USA) following the instructions in 11 µl reactions which consist of 1 µl PCR product or allelic ladder and the mixture of 9.6 µl Hi-Di Formamide and 0.4 µl GeneScan^®^-600 LIZ^®^ Size Standard v2.0. The loading mixture was first denatured at 95°C for 3 min, followed by cooling at 4°C for 3 min immediately. The results of STRs typing were identified by the GeneMapper ID 3.2 software (Applied Biosystems, Foster City, CA, USA). Deionized water and control DNA from human cell line 9948 were typed as negative and positive control, respectively.

### Statistical analysis

We calculated allelic frequencies, MP, PE, DP, and PIC using the corrected PowerStats v1.2 [30]. We evaluated Ho and He by making use of GenAlEx software version 6.503 [31] which could analyze a range of population genetic data. The test of HWE for each locus was performed by the Genepop v4.0.10 using the Markov chain algorithm. AMOVA was employed to calculate the inter-population differentiation by ARLEQUIN v3.5.1.2 software (http://cmpg.unibe.ch/software/arlequin3). On the basis of 14 overlapping STR loci, *Fst* values of pairwise populations were calculated with the Genepop v4.0.10. A PCA of eighteen populations was plotted by MVSP v3.1 software which could perform a variety of ordination and cluster analyses. A MDS plot was illustrated based on *Fst* values via the ‘R’ packages. Two phylogenic trees were constructed based on the allelic frequencies of the same 14 STRs by PHYLIP v3.6 software and *D*_A_ distances (obtained from the DISPAN program) by MEGA v5 software, respectively.

## SUPPLEMENTARY MATERIALS TABLE





## Abbreviations

STRs: short tandem repeats
HWE: Hardy-Weinberg equilibrium
MP: match of probability
PE: probability of exclusion
DP: discrimination power
PIC: polymorphic information content
Ho: observed heterozygosity
He: expected heterozygosity
CMP: combined match probability
CPE: combined probability of exclusion
AMOVA: analysis of molecular variance
*Fst*: fixation index
PCA: principal component analysis
MDS: multidimensional scaling
HLA: human leucocyte antigen
VNTR: variable number of tandem repeat
