# Supplementary material for: Phylogenic analysis and forensic genetic characterization of Chinese Uyghur group via autosomal multi STR markers
Source: Oncotarget. 2017 May 18;8(43):73837–45. doi: 10.18632/oncotarget.17992 (PMC5650305; doi:10.18632/oncotarget.17992)
Supplement: Supplementary file 1 [file oncotarget-08-73837-s001.pdf]

Phylogenic analysis and forensic genetic characterization of Chinese uyghur group via autosomal multi STR markers

SUPPLEMENTARY MATERIALS

Supplementary Table 1: The overall *Fst* distances among 18 compared populations based on the same 14 STR loci. See Supplementary_Table_1.
