# Supplementary material for: Phylogenic analysis and forensic genetic characterization of Chinese Uyghur group via autosomal multi STR markers
Source: Oncotarget. 2017 May 18;8(43):73837–45. doi: 10.18632/oncotarget.17992 (PMC5650305; doi:10.18632/oncotarget.17992)
Supplement: Supplementary file 2 [file oncotarget-08-73837-s002.doc]

Supplementary Table 1: The overall *Fst* distances among 18 compared populations based on the same 14 STR loci

| Populations | Spanish | Mexican | Portuguese | African American | Caucasian | Hispanics | Asian | Uyghur | Uyghur1 | Xibe | Tibetan | She | Yi | Hui | Beijing Han | Guangdong Han | Shaanxi Han |
| --- | --- | --- | --- | --- | --- | --- | --- | --- | --- | --- | --- | --- | --- | --- | --- | --- | --- |
| Mexican | 0.0287 |  |  |  |  |  |  |  |  |  |  |  |  |  |  |  |  |
| Portuguese | 0.0005 | 0.0271 |  |  |  |  |  |  |  |  |  |  |  |  |  |  |  |
| African American | 0.0186 | 0.0350 | 0.0184 |  |  |  |  |  |  |  |  |  |  |  |  |  |  |
| Caucasian | 0.0029 | 0.0275 | 0.0008 | 0.0188 |  |  |  |  |  |  |  |  |  |  |  |  |  |
| Hispanics | 0.0055 | 0.0169 | 0.0050 | 0.0157 | 0.0050 |  |  |  |  |  |  |  |  |  |  |  |  |
| Asian | 0.0187 | 0.0322 | 0.0157 | 0.0310 | 0.0164 | 0.0126 |  |  |  |  |  |  |  |  |  |  |  |
| Uyghur | 0.0071 | 0.0225 | 0.0066 | 0.0206 | 0.0073 | 0.0044 | 0.0058 |  |  |  |  |  |  |  |  |  |  |
| Uyghur1 | 0.0066 | 0.0231 | 0.0064 | 0.0207 | 0.0061 | 0.0042 | 0.0064 | 0.0009 |  |  |  |  |  |  |  |  |  |
| Xibe | 0.0170 | 0.0309 | 0.0151 | 0.0308 | 0.0159 | 0.0124 | 0.0028 | 0.0043 | 0.0047 |  |  |  |  |  |  |  |  |
| Tibetan | 0.0178 | 0.0323 | 0.0158 | 0.0318 | 0.0164 | 0.0133 | 0.0048 | 0.0054 | 0.0056 | 0.0024 |  |  |  |  |  |  |  |
| She | 0.0306 | 0.0410 | 0.0301 | 0.0510 | 0.0317 | 0.0261 | 0.0157 | 0.0173 | 0.0160 | 0.0108 | 0.0135 |  |  |  |  |  |  |
| Yi | 0.0195 | 0.0340 | 0.0187 | 0.0336 | 0.0204 | 0.0159 | 0.0046 | 0.0082 | 0.0079 | 0.0037 | 0.0056 | 0.0124 |  |  |  |  |  |
| Hui | 0.0126 | 0.0307 | 0.0114 | 0.0288 | 0.0126 | 0.0112 | 0.0024 | 0.0038 | 0.0037 | 0.0015 | 0.0028 | 0.0144 | 0.0032 |  |  |  |  |
| Beijing Han | 0.0199 | 0.0362 | 0.0182 | 0.0366 | 0.0194 | 0.0169 | 0.0026 | 0.0070 | 0.0078 | 0.0010 | 0.0019 | 0.0127 | 0.0031 | 0.0016 |  |  |  |
| Guangdong Han | 0.0198 | 0.0318 | 0.0166 | 0.0328 | 0.0179 | 0.0155 | 0.0018 | 0.0067 | 0.0071 | 0.0029 | 0.0050 | 0.0125 | 0.0039 | 0.0035 | 0.0032 |  |  |
| Shaanxi Han | 0.0165 | 0.0330 | 0.0157 | 0.0334 | 0.0172 | 0.0142 | 0.0030 | 0.0058 | 0.0056 | 0.0008 | 0.0024 | 0.0097 | 0.0035 | 0.0012 | 0.0000 | 0.0025 |  |
| Henan Han | 0.0183 | 0.0343 | 0.0170 | 0.0356 | 0.0186 | 0.0150 | 0.0022 | 0.0066 | 0.0067 | 0.0011 | 0.0030 | 0.0117 | 0.0029 | 0.0016 | -0.0002 | 0.0030 | -0.0001 |
